# The Relationship Between Physical Fitness Qualities and Sport-Specific Technical Skills in Female, Team-Based Ball Players: A Systematic Review

**DOI:** 10.1186/s40798-020-00245-y

**Published:** 2020-04-15

**Authors:** Jessica B. Farley, Joshua Stein, Justin W. L. Keogh, Carl T. Woods, Nikki Milne

**Affiliations:** 1 Faculty of Health Sciences and Medicine, Bond Institute of Health and Sport, Bond University, Robina, Australia; 2 Sports Performance Research Centre New Zealand, AUT University, Auckland, New Zealand; 3 Cluster for Health Improvement, Faculty of Science, Health, Education and Engineering, University of the Sunshine Coast, Sippy Downs, Australia; 4 Kasturba Medical College, Mangalore, Manipal Academy of Higher Education, Manipal, Karnataka India; 5 Institute for Health and Sport, Victoria University, Melbourne, Australia

**Keywords:** Women, Fitness attributes, Skill acquisition, Performance, Team sports

## Abstract

**Background:**

Understanding the relationships between physical fitness attributes and sport-specific technical skills may assist with the establishment of beneficial training interventions. The aim of this systematic review was to determine if physical fitness qualities were associated with performance of sport-specific technical skills in female, team-based ball players.

**Methods:**

Five databases (MEDLINE, EMBASE, SPORTDiscus, ProQuest, and Scopus) were searched for relevant studies up to April 15, 2019. Full-text articles investigating relationships between physical fitness and sport-specific technical skills in female, team-based ball players were included. Methodological quality of included studies was appraised using a modified Downs and Black checklist. Data synthesis was conducted to determine the summary of evidence based on the number of significantly associated relationships divided by the total relationships assessed. An individual relationship was defined as a reported result examining the association between a single physical fitness variable and a single sport-specific technical skill.

**Results:**

Data synthesis of grouped female ball players from 41 included studies revealed three clear associations: (i) body composition and defensive technical skills (19/29; 66%), (ii) agility and movement with a ball (4/6; 67%), and (iii) coordination and movement with a ball (3/5; 60%). An uncertain association exists between cardiorespiratory fitness and defensive technical skills (2/5; 40%). No association or an unknown conclusion (< 5 relationships reported) exists between all remaining physical fitness attributes and sport-specific technical skills. Sub-analyses revealed clear associations between body composition and throwing/shooting (using hands) in junior ball players (15/15; 100%) and with movement with a ball in elite (6/9; 67%) and senior (6/6; 100%) ball players. Three uncertain associations were found between offensive technical skills and agility in elite ball players (3/8; 38%), and with speed in elite (6/14; 43%) and junior (7/18; 39%) female ball players.

**Conclusion:**

There is limited evidence available to demonstrate relationships between physical fitness qualities and sport-specific technical skills in female, team-based ball players. This lack of association is possibly due to reductionist methods in available literature and a lack of research in female ball players globally.

**Registration:**

CRD42017065339 (PROSPERO on 12 May 2017).

## Key Points

- In female, team-based ball players, a relationship exists between (i) defensive technical skills and body composition, (ii) movement with a ball and agility, and (iii) movement with a ball and coordination. These findings may assist sport practitioners to enhance position-specific conditioning and talent development programs in female, team-based ball players.
- Most physical fitness components were not associated with sport-specific technical skills in female, team-based ball players.
- High-quality, holistic evidence, including a wider range of team-based ball sports, is needed to better understand the relationship and the role that physical fitness plays in combination with other attributes pertinent to sport-specific technical skills in female, team-based ball players.

## Background

Successful performance in team-based ball sports is commonly attributed to a unique combination of gifted and trained physical, technical, tactical, and psychosocial qualities [1, 2]. Measuring these multidimensional qualities could offer practitioners insight into game or sport demands [3, 4], prospective performance potential (i.e. talent identification) [5], and identify areas for continued player development [6, 7]. Further, longitudinal evaluation of these measured performance qualities is likely to assist practitioners in the effectiveness of training and rehabilitation interventions across different phases of the sport season (e.g. preseason, competition, and transition) [6, 7].

The physical preparation of ball players developed over several years is just one important factor in the success or failure of sporting outcomes [6]. Physical fitness is defined as a set of qualities that an individual has or develops relating to their ability to perform physical activity [8]. These measurable qualities commonly include the components of body composition, cardiorespiratory fitness, muscular strength, muscular endurance, flexibility, agility, balance, coordination, power, reaction time, and speed [8, 9]. More specific terms of physical fitness have been defined, with these physical fitness components further divided into two categories: (i) health-related physical fitness and (ii) performance-related physical fitness [8]. These physical fitness categories and their respective attributes can be considered inter-related [10], and depending upon the sport context, there may be some differences in the specific combination of which components are most required for success in a specified sport [11]. Thus, skill adaptation in sport-specific conditions augments one’s ability to produce optimal actions to enhance sport performance [10]. Therefore, for the purpose of this review, the physical fitness components will be classified together under the global term of ‘physical fitness’.

Physical fitness components have been shown to have a significant positive relationship with enhanced outcomes in physical activity, including sports participation [12]. There is a substantial amount of published research delineating the various physical fitness components required to successfully compete across team-based ball sports [13–18]. Additionally, there is a large quantity of work profiling the physical fitness qualities of different playing positions in various land-oriented, team-based ball sports, such as soccer [19–21], rugby league [22, 23], volleyball [24, 25], Australian football [26], and team handball [27, 28]. This position-specific, physical fitness profiling research can be of practical use for team sport practitioners, when attempting to optimise the specificity of training at the positional level to enhance the success of both the individual players and the team.

Physical fitness testing has also been used to discriminate elite players from their sub-elite peers, which offers a basis for the detection and identification of talent [7]. For example, a review by Lorenz and colleagues [29] describes specific performance characteristics seen in elite players of team-based ball sports, such as rugby and volleyball, and endurance-based sports, including swimming and running. Elite players often demonstrate superior power qualities relative to their sub-elite counterparts in field and court sports, which is likely to implicate speed and agility components [29]. However, physical fitness attributes including anthropometric characteristics, such as height, weight, and body fat percentage, are seemingly less sensitive in the identification of prospective performance potential in team-based ball players [29].

While players may require a wide variety of physical fitness attributes to meet the demands of game-play, another vital element required to successfully compete in sport includes the possession of sport-specific technical skills [30]. Sport-specific technical skills are considered to be actions involving a specific task or goal that require the coordination of multiple motor competencies relative to a time horizon and context [31]. Examples may include kicking a soccer ball to another player to move the ball down field or pitching a baseball to strike out an opponent. Given their centrality to success within sport, understanding the relationships between physical fitness attributes and sport-specific technical skills may assist with the establishment of beneficial training interventions.

The empirical literature has demonstrated sex differences in team-based ball sports performance [32–34]; however, minimal research has investigated the relationship between physical fitness and sport-specific technical skills in female populations. Consideration of sex-specific relationships may be integral when developing sport training regimes to ensure optimal player performance, especially due to the relative lack of research focused on female players. Therefore, the purpose of this systematic review was to identify and critically appraise the available literature to investigate if physical fitness is associated with performance of sport-specific technical skills in female, team-based ball players.

## Methods

### Registration

In accordance with the Preferred Reporting Items for Systematic Reviews and Meta-analyses (PRISMA) guidelines [35], this systematic review was registered with the International Prospective Register of Systematic Reviews (PROSPERO) on 12 May 2017 (registration number CRD42017065339).

### Data Sources

PROSPERO was initially searched for ongoing and previously registered systematic reviews to avoid duplication of research. Five scientific databases [MEDLINE (Ovid interface from 1946 to present), EMBASE (from 1947 to present), SPORTDiscus (from 1985 to present), ProQuest (from 1937 to present), and Scopus (from 1970 to present)] were searched for relevant studies up to April 15, 2019. Medical subject headings (MeSH) and text words were searched in all fields using syntax specific to each database.

### Search Strategy

A MEDLINE literature search strategy was developed with assistance from the primary author’s university faculty librarian with expertise in systematic review searching. The search strategy included search terms using MeSH and text words related to the concepts of the research question using the PICO (population, intervention, comparison/control, outcome) format: female, land-oriented, team-based ball players (P); physical fitness measures (I/C); and relationship with sport-specific technical skills (O) [36]. Four of the five contributing authors revised the final search strategy prior to conducting the search. The finalised MEDLINE search strategy (Online Resource 1) was modified to the syntax and subject headings, when appropriate, for the other four databases. Filters used to narrow results per inclusion criteria included ‘English’ and ‘journal article’.

### Eligibility Criteria

The inclusion of studies in this systematic review was determined using the following criteria:

#### Study Design

Original research studies of observational (prospective or retrospective cohort studies, case-control, cross-sectional, case series, or case reports) design were included. Interventional studies were included only if there was a comparison of baseline data between objective measures or if pre- and post-values of objective measures were reported. Intervention studies that did not meet these criteria and literature reviews were excluded.

#### Participants

For the premise of this review, land-oriented, team-based ball sports were categorised as invasion games, net/wall games, and striking/fielding games [37]. Therefore, studies were included that investigated a female population participating in any level of competition (youth, recreational, sub-elite, elite, etc.) in one of the following land-oriented, team-based ball sports: basketball, volleyball, cricket, baseball, softball, handball, netball, lacrosse, field hockey, or any football code (Australian football, Gaelic football, American football, flag football, soccer, futsal, indoor soccer, rugby union, rugby league, rugby sevens). Studies that investigated both male and female populations were included only if the subset of data for the female ball players were reported separately. Those studies investigating female ball players with a physical or mental disability were excluded, as inclusion of these data may provide different relationships to those of able-bodied players. Additionally, studies that examined male ball players only were excluded.

#### Intervention/Exposure

Studies were included if objective measures of physical fitness were performed. These included any measure that addressed one of the following physical fitness components: (i) agility (including change of direction tests), (ii) balance, (iii) body composition (including anthropometric characteristics), (iv) cardiorespiratory fitness (including ability to perform high-intensity exercise), (v) coordination, (vi) flexibility, (vii) muscular endurance, (viii) muscular strength, (ix) power, (x) reaction time, and (xi) speed (including speed endurance) (Table 1). All objective outcome measures of physical fitness were accepted and categorised into the physical fitness category most representative of the actions required to perform the test and/or the unit measures utilised according to definitions in Table 1. Studies that used physical fitness measures developed for a specific sport (e.g. Australian Football League agility test) or assessment tools encompassing multiple physical fitness measures (e.g. Bruininks-Oseretsky Test of Motor Proficiency, Second Edition) were also included. Studies that only examined sport-related qualities outside the physical health realm, such as psychological or behavioural attributes utilising patient-reported outcome measures, were excluded.

**Table 1:** Definitions of physical fitness components [9, 38]

Component	Definition
Agility	The capacity to rapidly move the whole body in different directions with speed and accuracy.^a^
Balance	The maintenance of equilibrium while the whole body is moving or stationary.
Body composition	Describes the human body’s relative amount of muscle, fat, bone, and other tissue.^b^
Cardiorespiratory fitness	Relates to the ability of the circulatory and respiratory systems of the human body to supply oxygen during large-muscle, dynamic exercise.
Coordination	Relates to the ability to use body parts to accurately and smoothly perform motor tasks.
Flexibility	The available range of motion at a joint.
Muscular endurance	The ability of a muscle or muscle group to remain contracted or to contract repeatedly without fatigue.
Muscular strength	The ability of a muscle to produce force.
Power	The rate one is able to exert maximal force.
Reaction time	The time elapsed between a stimulus and onset of movement to respond to it.
Speed	The ability to perform a skill or movement quickly.^c^

^a^Tests that involved a response to a stimulus [39] or change of direction speed [40] were considered under the global term of ‘agility’

^b^Anthropometric measures were included in the ‘body composition’ category [9]

^c^Tests that measured speed endurance were considered under the global component of ‘speed’

#### Sport-Specific Technical Skill Outcome

A sport-specific technical skill outcome was defined as an action due to a task or goal produced by coordinated motor abilities relative to a sport-specific context [31]. For observational studies to be included, statistical associations between a physical fitness exposure and performance outcome of a sport-specific technical skill were reported. For interventional studies to be included, the pre- and post-test values or treatment effect for a sport-specific technical skill outcome were reported. Studies that only examined global match performance outcomes of a sport (e.g. running distance in match-play or wins versus losses), rather than a sport-specific technical skill, were excluded.

#### Language

Articles published in English only were included in this review.

#### Other

Peer-reviewed journals containing accessible full-text articles only were included in this review. There was no date of publication restriction applied to this systematic search strategy, and electronic searches were date limited only by the electronic publications accessible in each database.

### Data Management

Literature search results were exported to an electronic reference management software program, EndNote (version X7, by Thomson Reuters). This software was used to store all references and identify duplicates. The evidence eligibility process, including screening titles and abstracts, categorising citations into inclusion and exclusion sets, and determining total number of records for synthesis, was conducted utilising the web-based software platform, Covidence (Covidence online systematic review platform, Veritas Health Innovation Ltd., Melbourne, Australia, www.covidence.org). Covidence is recommended by Cochrane to streamline the production of systematic reviews [41].

### Selection Process

Utilising the inclusion criteria, two reviewers (JBF, JS) independently screened the titles and abstracts generated by the search in Covidence. For records that appeared to meet the inclusion criteria, or for those citations where it was not clear, full-text manuscripts were obtained. The same two reviewers independently screened the full text against the eligibility criteria. Any discrepancies throughout the screening process were resolved in discussion by the two reviewers to reach consensus. Reasons for excluding records during the full-text screening stage were documented. The reviewers were not blinded to any recorded information, including study authors or journal titles.

### Data Extraction

Study data for each included study were extracted by one reviewer (JBF) and managed electronically in a spreadsheet using Microsoft Excel (version 2016). Data extracted included descriptive information of the study population, including number of participants, age, level of play, and sport identified. Data regarding the study design, physical fitness parameter(s) measured, sport-specific skill outcome(s) measured, statistical relationship results, and main findings reported regarding associations between physical fitness and sport-specific technical skills were also extracted.

### Critical Appraisal of Methodological Quality in Individual Studies

While relevant literature was published in intervention studies, the authors recognised that many included studies were observational in nature. As such, the methodological quality of studies eligible for review were critically appraised independently by two reviewers (JBF, JS) using a modified Downs and Black checklist [42]. When assessing observational studies, questions specific to intervention studies (items 4, 8, 14, 15, 19, 23, and 24) were removed and items 9, 13, and 22 were modified, with a total Downs and Black critical appraisal score out of 21 points (Table 2). The Downs and Black checklist [42] has previously been modified to suit methodological quality assessment of other study designs in health science research [43–46]. All original 27 items remained as intended for intervention studies with a total critical appraisal score out of 28 points [42], as the scoring of item 27 was modified to a dichotomous scale (yes = 1, no = 0) as previously reported [43, 44, 47].

**Table 2:** Modified Downs and Black critical appraisal checklist applied to observational studies (adapted from Downs and Black [42])

Item #	Question
1	Is the hypothesis/aim/objective of the study clearly described?
2	Are the main outcomes to be measured clearly described in the introduction or methods section?
3	Are the characteristics of the participants included in the study clearly described?
4	Removed.
5	Are the distributions of principal confounders clearly described?
6	Are the main findings of the study clearly described?
7	Does the study provide estimates of the random variability in the data for the main outcome?
8	Removed.
9^a^	Have the characteristics of patients lost to follow-up been described or did the study have any participant losses?
10	Have actual probability values been reported for the main outcomes, except where the probability value is < 0.001?
11	Were the subjects asked to participate in the study representative of the entire population from which they were recruited?
12	Were those subjects who were prepared to participate representative of the entire population from which they were recruited?
13^a^	Were the staff, places, and facilities where the participants were treated or where the testing was performed representative of the exams/treatment the majority would receive?
14	Removed.
15	Removed.
16	If any of the results of the study were based on ‘data dredging’ (i.e. ‘data fishing’), was this made clear?
17	In trials and cohort studies, did the analyses adjust for different lengths of follow-up of participants, or in case-control studies, was the time period between the intervention and the outcome the same for cases and controls?
18	Were the statistical tests used to assess the main outcomes appropriate?
19	Removed.
20	Were the main outcome measures used accurate (valid and reliable)?
21	Were the participants in different intervention groups (trials and cohort studies) or were the cases and controls (case-control studies) recruited from the same population?
22^a^	Were study subjects recruited over the same period of time?
23	Removed.
24	Removed.
25	Was there adequate adjustment for confounding in the analyses from which the main findings were drawn (e.g. the distribution of known confounders that differed between groups was taken into account in the analysis)?
26	Were losses of patients to follow-up taken into account?
27	Did the study have sufficient power to detect a clinically important effect where the probability value for a difference being due to chance was less than 5%?

Scoring criteria [42]: items 1–3, 6, 7, 9–13, 16–18, 20–22, 25–27: yes = 1, unable to determine/no = 0; item 5: yes = 2, partially = 1, no = 0

^a^Indicates item number was modified

In order to standardise the Downs and Black methodological quality scoring across intervention and observational studies, the rating scale proposed by Kennelly [48] was applied. Raw critical appraisal scores were used to grade the overall methodological quality of each observational study as either poor (≤ 10), fair (11–14), or good (≥ 15). This method is a similar approach to previously published reviews [43, 44]. The modified rating scale utilises the same ratios from the rating scale originally proposed by Kennelly [48] of poor (≤ 14), fair (15–19), and good (≥ 20), which was applied to included intervention studies. Risk of bias (ROB) was examined by identifying the internal validity subset items (bias and confounding) on the Downs and Black [42] checklist that were pertinent to assessing ROB in observational studies [49]. These relevant Downs and Black [42] items included items 16, 18, 20, 21, 22, and 25. Each item was given a score of 1 for ‘yes’ or 0 for ‘no/unable to determine’. Low ROB was determined by a total score greater than or equal to 4/6 (67%). The critical appraisal and ROB analyses were performed by two reviewers (JBF, JS) independently with any discrepancies resolved by a third reviewer (NM).

### Data Synthesis

The quality of evidence for all outcomes was synthesised using methods initially described by Sallis and colleagues [50] and subsequently applied to reviews investigating relationships between objective health outcomes (including physical fitness measurements) and physical activity [51, 52] and academic performance [43]. For the purpose of this review, a relationship was defined as a reported result examining the association between a single physical fitness variable and a single sport-specific technical skill. Therefore, a particular study may demonstrate multiple relationships if numerous physical fitness variables were examined against one or more sport-specific technical skill. Repeated data reported in multiple studies from the same source were only accounted for once in the data synthesis.

The cumulative strength of the body of evidence for each category of sport-specific technical skill and its relationship with physical fitness was classified utilising a coding system adapted from Sallis and colleagues [50]. The extracted data was synthesised using a summary conclusion that was calculated as a percentage based on the number of significantly associated relationships divided by the total number of relationships investigated. This percentage was then used to classify the cumulative strength of evidence and to underpin a practical interpretation of results based on the following: ≤ 33% indicated ‘no association’, 34–59% revealed ‘uncertain association’, or a ‘clear association’ shown by ≥ 60%, similar to that previously reported [43, 50–52]. The statistical association direction reported for each relationship was noted as either positive ‘+’, negative ‘−’, or identified as having both positive and negative ‘+/−’ statistical association directions to assist in the practical interpretation of the evidence. Given the plethora of ways to measure various physical fitness and sport-specific technical skill outcomes, the methods regarding how variables were measured were considered when interpreting the statistical association direction to enhance understanding of the summary of evidence to draw practical interpretations. For example, one relationship between a coordination measure and a sport-specific technical skill could reveal a negative statistical association direction, whereas another relationship could report a positive statistical association direction. When examining how the coordination attributes were measured, the first may report the outcome as time (i.e. faster, or lower number, is better) and the other could be measured in counts (i.e. higher number is better). Despite the contrast in statistical association direction in this example, the practical interpretation concluded would be better coordination performance indicates better sport-specific technical skill performance. Additionally, if more than one but less than five relationships were reported on a physical fitness outcome, the summary conclusion was deemed as ‘unknown’ due to insufficient evidence found. Conclusions for this review were developed from the summative synthesis of studies with a Kennelly [48] rating of ‘fair’ or ‘good’ with low ROB.

The strength of the correlation data between physical fitness attributes and sport-specific technical skill performance was interpreted utilising the following rating scale: *r* = 0.00–0.19 (very weak), *r* = 0.20–0.39 (weak), *r* = 0.40–0.59 (moderate), *r* = 0.60–0.79 (strong), and *r* = 0.80–1.0 (very strong) [53]. If studies did not report any statistical correlation results between some of the potential relationships, then these relationships were not accounted for in the data synthesis, as their association was deemed unable to determine. When associations were reported, however without evidence of significance (e.g. *p* values or a direct statement regarding significance), these measures were deemed as not associated with the reported technical skill. To examine the impact on the summary conclusion and practical interpretation, a sensitivity analysis was conducted by including those associations that had a strength of correlation deemed as moderate, strong, or very strong, even if these values were reported without the associated level of statistical significance. Additionally, sub-analyses were conducted to investigate the impact of competition level (elite versus non-elite), age (senior ≥ 18 years old versus junior < 18 years old), and skeletal maturity (≥ 15 years old versus < 15 years old) on the relationships between physical fitness and sport-specific technical skills. This involved the same data synthesis process as described above for each sub-category. Where information regarding competition level and mean age of the study participants were not reported, or if the study included combined sub-category results, these relationships were excluded from the sub-analysis as the required data from the sub-category could not be isolated.

For studies that implemented an alternative statistical analysis to examine associations between combined physical fitness attributes and sport-specific technical skill performance, such as a canonical correlation, a critical narrative synthesis was performed to synthesise key findings. A meta-analysis was not performed due to the heterogeneity amongst the included studies in this review regarding their study design, physical fitness variables assessed, and sport-specific technical skill outcome measures.

## Results

### Study Selection

**Fig. 1:**
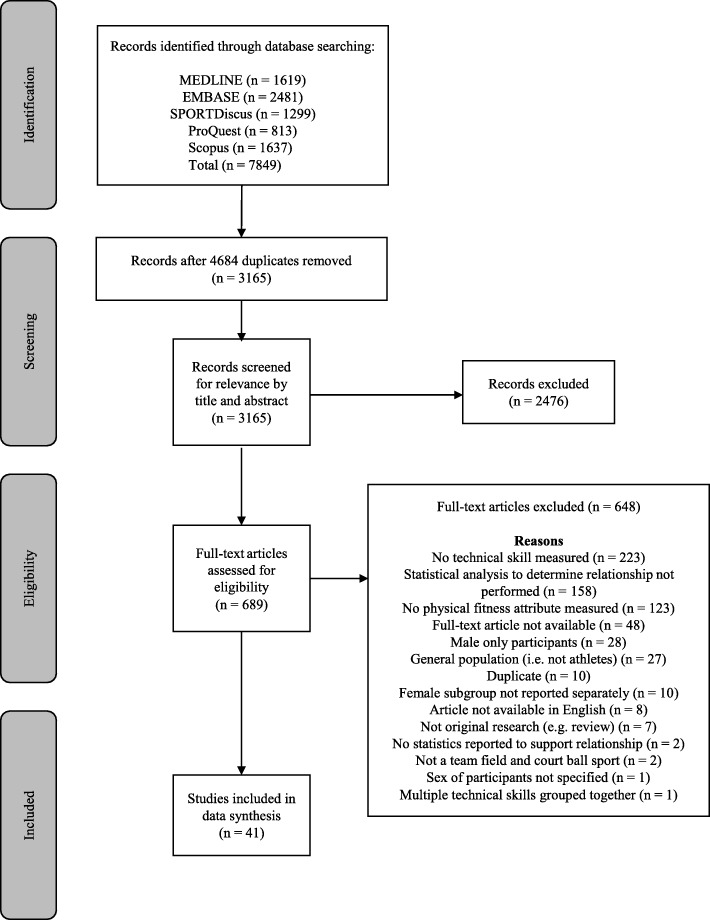
PRISMA flow diagram outlining the search, screening, and selection review process [35]

The search of five databases revealed 7849 records, with 3165 studies available for review after duplicates were removed (Fig. 1). Following screening of title and abstract and subsequent full-text evaluation, 41 studies were included in the review.

### Study Characteristics

Key data extracted for this review are outlined in Online Resource 2. Of the 41 studies included in the review, only one study was interventional in design [54], with the remaining observational in nature. Research in handball [54–68] was the most frequently included team-based, ball sport followed by volleyball [69–80]. Other sports investigated included soccer [81–86], basketball [87–90], netball [91, 92], lacrosse [93], and softball [94]. Female participant sample sizes ranged from 10 to 246, with ball players involved in varied competitions from non-elite (15 studies, 37%) to elite/national game-play level (26 studies, 63%). The age range of female ball players was 12 to 27 years, with 15 studies (37%) involving youth players. Female team-based, ball players from Spain, Croatia, Estonia, and Norway collectively represented 46% of studies included in this review. Five studies (12%) did not report the participants’ country of origin, with the remaining 42% of studies spanning 13 other countries across four continents (Asia, Australia, Europe, and North America).

A range of methods were used to measure the 11 physical fitness components. Agility qualities were investigated by 13 (32%) studies [55, 56, 61, 71, 72, 79, 81, 84, 85, 88–90, 92]. Balance ability was measured in only four (10%) studies [57, 72, 83, 93]. Body composition was measured in 27 (66%) of the included studies [56, 58, 60, 61, 63–67, 69, 72, 73, 75–82, 85–87, 89–92]. Nine (22%) included studies examined cardiorespiratory fitness attributes [58, 61, 65, 68, 79, 81, 85, 88, 92]. Four (10%) studies examined coordination abilities [55, 56, 61, 71]. Flexibility was measured by five (12%) of the included studies [66, 72, 79, 90, 91]. Upper and/or lower body muscular strength and muscular endurance were assessed in 18 (44%) [54, 58, 59, 62, 65–68, 72, 74, 75, 80, 81, 88, 90, 91, 93, 94] and three (7%) [72, 79, 90] of the included studies, respectively. Nineteen (46%) studies measured power produced by the extremities, such as via a countermovement jump or medicine ball throw [56, 58–62, 65, 68, 70–72, 79–81, 85, 88–90, 92]. Reaction time was the least investigated physical fitness measure, with only two studies (5%) assessing this attribute [77, 79]. Finally, 12 (29%) studies included speed measures in their analysis [56, 58, 61, 65, 68, 81, 84, 85, 88–90, 92].

### Methodological Quality of Included Studies

Table 3 demonstrates the critical appraisal score for each of the included studies in the review. Cohen’s kappa analysis initially revealed a moderate level of agreement between the two reviewers (JBF, JS) (*κ* = 0.572, *p* < 0.0005). After a process of consensus, 100% agreement was achieved between reviewers regarding critical appraisal scores. The breakdown of included studies having ‘good’, ‘fair’, and ‘poor’ methodological quality based on the Kennelly [48] rating were 10 (24%), 25 (61%), and 6 (15%), respectively. Seven (17%) studies were considered to have high ROB. Noteworthy limitations amongst the studies included the following: participants were not representative of the entire population from which they were recruited, no adequate adjustment for confounding variables, and only three studies demonstrated adequate power analysis for their study sample [62, 63, 92]. There was also a limited number of studies reporting actual probability values for the main outcomes.

**Table 3:** Critical appraisal scores, Kennelly ratings [48], and risk of bias assessment based on modified Downs and Black [42]

Study author (year)	Critical appraisal score (out of 21)	Kennelly rating	Risk of bias
Bojić and Pavlović (2015) [55]	11	Fair	Low
Brooks et al. (2013) [81]	7	Poor	High
Čavala et al. (2008) [56]	12	Fair	Low
Dyer et al. (2018) [87]	12	Fair	Low
Elliot and Smith (1983) [91]	12	Fair	Low
Eriksrud et al. (2019) [57]	10	Poor	Low
Fort-Vanmeerhaeghe et al. (2016) [88]	16	Good	Low
Garcia-Gil et al. (2018) [89]	15	Good	Low
Granados et al. (2008) [58]	14	Fair	Low
Granados et al. (2013) [59]	14	Fair	Low
Grgantov et al. (2007) [69]	10	Poor	High
Hoff and Almåsbakk (1995) [54]^a^	14	Poor	Low
Ikeda et al. (2018) [70]	14	Fair	High
Jelaska et al. (2015) [82]	12	Fair	Low
Kaminski et al. (2007) [83]	14	Fair	High
Karadenizli (2016) [60]	14	Fair	Low
Katić et al. (2007) [61]	7	Poor	High
Katić et al. (2006) [71]	12	Fair	Low
Kutlu et al. (2017) [84]	15	Good	Low
Marsh et al. (2010) [93]	14	Fair	Low
McGhie et al. (2018) [62]	15	Good	High
Melrose et al. (2007) [72]	15	Good	Low
Mielgo-Ayuso et al. (2015) [73]	15	Good	Low
Moss et al. (2015) [63]	14	Fair	Low
Mujika et al. (2009) [85]	14	Fair	Low
Perroni et al. (2018) [86]	14	Fair	Low
Piscitelli et al. (2016) [64]	15	Good	Low
Pugh et al. (2001) [94]	10	Poor	High
Ramos et al. (2019) [90]	12	Fair	Low
Saavedra et al. (2018) [65]	13	Fair	Low
Sattler et al. (2015) [74]	16	Good	Low
Sattler et al. (2016) [75]	15	Good	Low
Schwesig et al. (2016) [66]	15	Good	Low
Stamm (2004) [76]	12	Fair	Low
Stamm et al. (2005) [77]	13	Fair	Low
Stamm et al. (2003) [79]	14	Fair	Low
Stamm et al. (2001) [78]	14	Fair	Low
Tissera et al. (2019) [92]	12	Fair	Low
Valadés et al. (2016) [80]	14	Fair	Low
van den Tillaar and Ettema (2004) [67]	12	Fair	Low
Wagner et al. (2019) [68]	11	Fair	Low

For all observational studies, a modified Kennelly rating was determined by raw critical appraisal score (out of 21) to assess the overall methodological quality of each study as either poor (≤ 10), fair (11–14), or good (≥ 15). Risk of bias rating, regardless of study design, was determined by internal validity subset items on the Downs and Black score (out of 6) as either low (≥ 4) or high (≤ 3)

^a^Intervention study; therefore, Kennelly rating determined by raw critical appraisal score (out of 28) was utilised to assess the overall methodological quality as either poor (≤ 14), fair (15–19), or good (≥ 20)

### Individual Relationships Between Physical Fitness Attributes and Sport-Specific Technical Skills

Sensitivity analysis of the data synthesis including results from relationships reported without evidence of significance revealed no change in the overall summary conclusion and practical interpretation of results. Sub-analyses regarding the impact of competition level (elite versus non-elite), age (senior ≥ 18 years old versus junior < 18 years old), and skeletal maturity (≥ 15 years old versus < 15 years old) are reported where a change from the grouped results occurred, except when an ‘unknown’ result due to insufficient evidence (i.e. more than one and less than five relationships) occurred.

#### Throwing and Shooting (Hand-Based) Sport-Specific Technical Skills

A total of 16 studies investigated associations between physical fitness outcomes and team-based ball sport throwing or shooting (hand-based) technical skills. Thirteen (81%) studies focused on handball throwing skills [54, 55, 57–60, 62–68], with the remaining three studies investigating softball [94], lacrosse [93], and netball [91] throwing/shooting (hand-based) technical skills, respectively. Thirteen (81%) studies examined throwing velocity [54, 57–60, 63–68, 93, 94], four (25%) studies measured throwing/shooting (hand-based) accuracy or precision [55, 57, 91, 93], and two (13%) studies used throwing jump height to examine sport-specific technical skill [62, 68]. Four (25%) studies were considered to have high ROB and/or poor methodological quality score [54, 57, 62, 94].

Table 4 provides a data synthesis of the associations between physical fitness attributes and throwing/shooting (hand-based) sport-specific technical skills. The number of relationships assessed from each study is reported as either demonstrating a significant or not significant association with technical skill, with the study reference number indicated. The summary conclusion outcome is reported with the number of significantly associated relationships divided by the total number of relationships investigated (*n*/*N*) and reported as a percentage. A total of 657 relationships between all physical fitness measures and throwing/shooting (hand-based) sport-specific technical skills were reported from the 12 studies demonstrating fair/good methodological quality with low ROB. Data synthesis revealed the level of evidence as ‘no association’ between all physical fitness components investigated and throwing/shooting (hand-based) sport-specific technical skills in female ball players, as less than 33% of relationships demonstrated a significant association (Table 4).

**Table 4:** Relationships between physical fitness components and throwing/shooting (hand-based) sport-specific technical skills from studies with fair or good methodological quality with low ROB

Physical fitness measures	Associated with technical skill		Not associated with technical skill	Summary conclusion	
	# relationships assessed from each study	Statistical association	# relationships assessed from each study	*n*/*N* outcome (%)	Practical interpretation
Agility measures	1 [55]	+	7 [55]	1/8 (13%)	No association
Balance measures	1 [93]	+	7 [93]	1/8 (13%)	No association
Body composition measures	3 [67], 4 [60], 4 [65], 8 [64], 15 [63]	+/−	1 [60], 2 [65], 6 [66], 17 [91], 70 [58], 244 [64]	34/374 (9%)	No association
Cardiorespiratory fitness measures	0	N/A	2 [65], 14 [58]	0/16 (0%)	No association
Coordination measures	1 [55]	−	2 [93], 19 [55]	1/22 (5%)	No association
Flexibility measures	1 [91]	+/−	1 [91], 20 [66]	1/22 (5%)	No association
Muscular strength measures	1 [65], 1 [67], 1 [68]	+	1 [65], 2 [59], 2 [93], 4 [82], 14 [58], 16 [66]	3/42 (7%)	No association
Power measures	1 [68], 3 [59], 4 [58], 4 [65]	+	2 [60], 11 [59], 122 [58]	12/147 (8%)	No association
Speed measures	2 [65]	−	2 [65], 14 [58]	2/18 (11%)	No association

Coding: *n*/*N* number of significantly associated relationships divided by total number of relationships. *#* number of relationships reported with study reference number in brackets. Statistical association: *+* positive statistical correlations, *−* negative statistical correlations, *+/−* includes both positive and negative statistical correlations. Practical interpretation: no association (≤ 33% of total relationships are significantly associated). Note: Relationships removed from data synthesis due to poor methodological study quality and/or high risk of bias included the following categories: balance (36 relationships [57]); muscular strength (2 relationships [54], 6 relationships [62], 3 relationships [94])

Sub-analysis examining the impact of senior (≥ 18 years old) versus junior (< 18 years old) study participants revealed no change in the above findings for senior ball players. However, sub-analysis concluded a ‘clear association’ (15/15; 100%) between body composition and throwing/shooting (using hands) sport-specific technical skills when junior ball players were examined separately. Above-average stature and mass, lower body fat percentage, and larger lower extremity girths were statistically associated (*r* = −0.34–0.65, *p* < 0.001−0.02) with better handball throwing performance. Sub-analyses investigating the impact of competition level and skeletal maturity revealed no change in reported results. No relationships were reported between throwing/shooting (hand-based) sport-specific technical skills and the physical fitness components of muscular endurance and reaction time.

#### Kicking and Shooting (Foot-Based) Sport-Specific Technical Skills

Three studies investigated associations between physical fitness attributes and kicking or shooting (foot-based) sport-specific technical skills. One study examined ball velocity [81], while the other two studies used goal success or number of passing/shooting points to examine soccer kicking technical skill [84, 86]. One (33%) study was considered to have high ROB [81]. Therefore, from the two studies with fair/good methodological quality with low ROB, a total of 16 relationships examined the association between physical fitness attributes and soccer kicking/shooting technical skills. Data synthesis revealed ‘no association’ (0/12; 0%) between body composition attributes, including weight, height, and BMI measures, and kicking/shooting (foot-based) sport-specific technical skills. Despite 100% of the relationships demonstrated an association between kicking/shooting (foot-based) sport-specific technical skills and both speed (1/1 relationship) and agility (3/3 relationships), the summary of evidence determined by data synthesis was ‘unknown’, as less than five relationships were reported for the respective physical fitness components. Sub-analyses examining the impact of competition level, age, and skeletal maturity revealed no change in reported findings. No relationships were reported between kicking and shooting (foot-based) sport-specific technical skills and the following physical fitness components: cardiorespiratory fitness, muscular strength, muscular endurance, flexibility, balance, coordination, power, and reaction time.

#### Movement with a Ball (Using Hands or Feet) Sport-Specific Technical Skills

Five studies investigated associations between physical fitness outcomes and movement with a ball (using hands or feet) sport-specific technical skills. Four studies focused on dribbling or juggling skills with a soccer ball [84–86] and basketball [89], with the remaining study investigated running speed with a handball [55]. All studies were considered to have fair/good methodological quality with low ROB.

Table 5 provides a data synthesis of the associations between physical fitness attributes and movement with a ball (using hands or feet) sport-specific technical skills. A total of 46 relationships were investigated from the five studies with fair/good methodological quality with low ROB. Due to the different methods used for assessment, four relationships found a significant weak to strong positive statistical association (*r* = 0.39–0.61, *p* = 0.001–0.02) with soccer dribbling and agility, whereas three relationships reported a significant weak negative statistical association (*r* = − 0.21 to − 0.33, *p* values not reported) between coordination abilities and movement with a handball. Despite the contrast in statistical direction of association, a higher coordination test score indicated better (faster) performance of running with a ball. Therefore, ball players who performed better in agility or coordination measures had better movement with a ball (using hands or feet) performance outcomes (Table 5).

**Table 5:** Relationships between physical fitness components and movement with a ball (using hands or feet) sport-specific technical skills from studies with fair or good methodological quality with low ROB

Physical fitness measures	Associated with technical skill		Not associated with technical skill	Summary conclusion	
	# relationships assessed from each study	Statistical association	# relationships assessed from each study	*n*/*N* outcome (%)	Practical interpretation
Agility measures	1 [85], 3 [84]	+	2 [55]	4/6 (67%)	Clear association (better agility test scores = better movement with a ball outcome)
Body composition measures	3 [86], 6 [89]	+/−	3 [85], 18 [86]	9/30 (30%)	No association
Cardiorespiratory fitness measures	0	N/A	1 [85]	0/1 (0%)	Unknown
Coordination measures	3 [55]	−	2 [55]	3/5 (60%)	Clear association (better coordination test scores = better movement with a ball outcome)
Power measures	0	N/A	2 [85]	0/2 (0%)	Unknown
Speed measures	1 [84]	+	1 [85]	1/2 (50%)	Unknown

Coding: *n*/*N* number of significantly associated relationships divided by total number of relationships. *#* number of relationships reported with study reference number in brackets. Statistical association: *+* positive statistical correlations, *−* negative statistical correlations, *+/−* includes both positive and negative statistical correlations. Practical interpretation: clear association (≥ 60% of total relationships are significantly associated), no association (≤ 33% of total relationships are significantly associated), unknown conclusion (< 5 total relationships reported)

Data synthesis concluded ‘no association’ between body composition physical fitness qualities and movement with a ball (using hands or feet) sport-specific technical skills in the grouped analysis, as only 30% (9/30) of the relationships assessed were significant (Table 5). ‘Unknown’ conclusions were found between movement with a ball (using hands or feet) sport-specific technical skills and the physical fitness components of cardiorespiratory fitness (0/1; 0%), power (0/2; 0%), and speed (1/2; 50%), as less than five relationships were assessed for each physical fitness attribute separately (Table 5).

Sub-analysis revealed a ‘clear association’ (6/9; 67%) between body composition and movement with a ball (using hands or feet) sport-specific technical skills in female elite ball players. Six relationships revealed significant weak to strong (*r* = − 0.66–0.62, *p* < 0.05) associations between body composition measures, such as height, body mass, BMI, arm span, and body fat percentage, and movement with a soccer ball. Thus, elite female ball players with greater body composition measures (e.g. taller and heavier) performed worse on soccer dribbling tests. Similar ‘clear association’ findings (6/6; 100%) were found between body composition attributes and movement with a ball (using hands or feet) sport-specific technical skills in senior ball players. Sub-analysis revealed no change in results when impact of skeletal maturity was examined. No relationships were reported between movement with a ball (using hands or feet) sport-specific technical skills and physical fitness components of muscular strength, muscular endurance, flexibility, balance, or reaction time.

#### Heading Sport-Specific Technical Skill

Two soccer studies [83, 86] examined associations between physical fitness attributes and soccer headers (sport-specific technical skill), with one (50%) study considered to have high ROB [83]. Data synthesis revealed ‘no association’ (2/6; 33%) between body composition variables, including height, weight, and BMI, and soccer headers. The summary conclusion between game heading and balance was unable to be determined (0/0; 0%), as there were no studies with a low ROB investigating this relationship. Sub-analyses examining the impact of competition level, age, and skeletal maturity revealed no change in reported findings. No relationships were reported between soccer heading technical skill and the following physical fitness components: cardiorespiratory fitness, muscular strength, muscular endurance, flexibility, agility, coordination, power, reaction time, and speed.

#### Offensive Sport-Specific Technical Skills

Twelve studies investigated associations between physical fitness attributes and offensive sport-specific technical skills. Seven (58%) studies focused on volleyball offensive technical skills, such as spike jump and serve [70, 72, 73, 75, 77, 79, 80]. Four (33%) studies investigated basketball offensive technical skills, including assists and points [87–90], with the remaining study examining successful netball catches and passes [92]. One (8%) study was considered to have high ROB [70].

Table 6 provides a data synthesis of the relationships reported between offensive sport-specific technical skills and physical fitness attributes. From the 11 studies with fair/good methodological quality with low ROB, a total of 345 relationships were investigated. Data synthesis concluded ‘no association’ between offensive sport-specific technical skills and the following physical fitness components: agility (3/19; 16%), body composition (27/152; 18%), cardiorespiratory fitness (3/9; 33%), flexibility (0/9; 0%), muscular endurance (0/7; 0%), muscular strength (1/20; 5%), power (11/69; 16%), reaction time (2/36; 6%), and speed (7/22; 32%) (Table 6). An ‘unknown’ (1/2; 50%) result between balance measures and offensive sport-specific technical skills was determined, as less than five relationships were reported (Table 6).

**Table 6:** Relationships between physical fitness components and offensive sport-specific technical skills from studies with fair or good methodological quality with low ROB

Physical fitness measures	Associated with technical skill		Not associated with technical skill	Summary conclusion	
	# relationships assessed from each study	Statistical association	# relationships assessed from each study	*n*/*N* outcome (%)	Practical interpretation
Agility measures	1 [88], 2 [90]	−	1 [88], 3 [79], 4 [72], 4 [89], 4 [92]	3/19 (16%)	No association
Balance measures	1 [72]	+	1 [72]	1/2 (50%)	Unknown
Body composition measures	1 [80], 1 [92], 2 [72], 7 [89],16 [73]	+/−	10 [87], 14 [73], 14 [90], 15 [92], 17 [80], 25 [89], 30 [72]	27/152 (18%)	No association
Cardiorespiratory fitness measures	1 [79], 1 [92], 1 [88]	+/−	2 [79], 1 [88], 3 [92]	3/9 (33%)	No association
Flexibility measures	0	N/A	2 [90], 3 [79], 4 [72]	0/9 (0%)	No association
Muscular endurance measures	0	N/A	2 [72], 2 [90], 3 [79]	0/7 (0%)	No association
Muscular strength measures	1 [72]	+	2 [90], 3 [72], 4 [75], 4 [88], 6 [80]	1/20 (5%)	No association
Power measures	1 [79], 1 [88], 2 [80], 2 [90], 5 [92]	+	4 [72], 4 [89], 7 [88], 8 [79], 8 [90], 11 [92], 16 [80]	11/69 (16%)	No association
Reaction time measures	2 [77, 79]	+/−	34 [77, 79]	2/36 (6%)	No association
Speed measures	1 [92], 2 [90], 4 [88]	+/−	4 [88], 4 [89], 7 [92]	7/22 (32%)	No association

Coding: *n*/*N* number of significantly associated relationships divided by total number of relationships. *#* number of relationships with study reference number in brackets. Statistical association: *+* positive statistical correlations, *−* negative statistical correlations, *+/−* includes both positive and negative statistical correlations. Practical interpretation: no association (≤ 33% of total relationships are significantly associated), unknown conclusion (< 5 total relationships reported). Note: Relationships removed from data synthesis due to poor methodological study quality and/or high risk of bias included the following category: power (2 relationships [54])

Upon sub-analysis, three relationships demonstrated significant very weak to strong (*r* = − 0.176 to − 0.701, *p* < 0.05) negative associations between offensive sport-specific technical skills and agility measures in elite ball players. In practical terms, better agility performance scores indicated better offensive sport-specific technical skill performance; however, this association is ‘uncertain’, as these findings only represent 38% (3/8) of the total relationships investigated in elite ball players. An ‘uncertain association’ was also revealed between offensive sport-specific technical skills and speed in junior (7/18; 39%) and elite (6/14; 43%) ball players. Similar to the agility results, all but one relationship demonstrated a significant very weak to strong (*r* = − 0.019 to − 0.676, *p* < 0.05) negative association between offensive sport-specific technical skills and speed outcomes. Thereby, faster sprinting times were related to better offensive sport-specific technical skill performance. Sub-analysis investigating the impact of skeletal maturity revealed no change in reported results. No relationships were investigated between offensive sport-specific technical skills and coordination physical fitness measures.

#### Defensive Sport-Specific Technical Skills

Eight studies investigated associations between defensive sport-specific technical skills and physical fitness attributes. Four (50%) studies focused on basketball defensive skills, such as rebounds and steals [87–90], whereas three (38%) studies examined volleyball defensive skills, such as block jump and reception [75, 77, 79]. One (13%) study investigated defensive time in a game-performance handball skill test [68]. All studies were considered to have fair/good methodological quality with low ROB.

Table 7 provides a data synthesis of the associations between physical fitness attributes and defensive sport-specific technical skills. Body composition measures, such as height, weight, and arm or hand span, demonstrated significant weak to strong (*r* = 0.360–0.742, *p* < 0.05) positive statistical associations (19/29; 66%) with defensive sport-specific technical skills. In other words, ball players who were taller, heavier, and had larger anthropometric measurements, such as hand span, arm span, and arm and thigh circumference, demonstrated better performance of defensive sport-specific technical skills.

**Table 7:** Relationships between physical fitness components and defensive sport-specific technical skills from studies with fair or good methodological quality with low ROB

Physical fitness measures	Associated with technical skill		Not associated with technical skill	Summary conclusion	
	# relationships assessed from each study	Statistical association	# relationships assessed from each study	*n*/*N* outcome (%)	Practical interpretation
Agility measures	1 [79], 1 [88]	−	1 [79], 1 [90], 2 [88], 2 [89]	2/8 (25%)	No association
Body composition measures	5 [90], 14 [89]	+	2 [89], 2 [90], 6 [87]	19/29 (66%)	Clear association (taller, heavier ball players with larger body composition measurements = better defensive skill performance)
Cardiorespiratory fitness measures	1 [79], 1 [88]	+/−	1 [79], 2 [88]	2/5 (40%)	Uncertain association
Flexibility measures	1 [79]	+	1 [79], 1 [90]	1/3 (33%)	Unknown
Muscular endurance measures	0	N/A	1 [90], 2 [79]	0/3 (0%)	Unknown
Muscular strength measures	1 [90]	+	4 [75], 6 [88]	1/11 (9%)	No association
Power measures	3 [90]	+	2 [89], 2 [90], 6 [79], 12 [88]	3/25 (12%)	No association
Reaction time measures	3 [77, 79]	+/−	21 [77, 79]	3/24 (13%)	No association
Speed measures	3 [88]	−	1 [68], 1 [90], 2 [89], 9 [88]	3/16 (19%)	No association

Coding: *n*/*N* number of significantly associated relationships divided by total number of relationships. *#* number of relationships reported with study reference number in brackets. Statistical association: *+* positive statistical correlations, *−* negative statistical correlations, *+/−* includes both positive and negative statistical correlations. Practical interpretation: clear association (≥ 60% of total relationships are significantly associated), uncertain association (34–59% of total relationships are significantly associated), no association (≤ 33% of total relationships are significantly associated), unknown conclusion (< 5 total relationships reported)

Two relationships demonstrated conflicting significant moderate (*r* = − 0.526–0.564, *p* < 0.05) associations between cardiorespiratory fitness and defensive sport-specific technical skills (Table 7). One relationship revealed worse cardiorespiratory fitness scores indicated better defensive sport-specific technical skill performance, whereas the other relationship demonstrated the opposite practical interpretation. As these results represented only 40% of the total relationships investigated, the summary conclusion was considered as an ‘uncertain association’ by data synthesis (Table 7). The data synthesis revealed ‘no association’ between defensive sport-specific technical skills and agility (2/8; 25%), muscular strength (1/11; 9%), power (3/25; 12%), reaction time (3/24; 13%), and speed (3/16; 19%) physical fitness attributes. Sub-analyses examining the impact of competition level, age, and skeletal maturity revealed no change in reported findings. No relationships were reported between defensive sport-specific technical skills and balance or coordination physical fitness components.

### Combined Physical Fitness Attributes and Association with Sport-Specific Technical Skills

Twelve (29%) of the included studies used a regression or canonical statistical analysis to examine the association between combined physical fitness components and sport-specific technical skills in female team, ball players. Nine of these studies examined technical skills specific to volleyball [69, 71, 74–80]. Isokinetic strength variables for knee flexors and extensors were found to be significant predictors of technical jumping performance in volleyball for elite senior female ball players (Can *R* = 0.46–0.65, *p* = 0.00–0.01), with a greater relationship reported with the defensive block jump [74, 75]. Specifically, performance in the block jump was predominantly contributed to by concentric strength of the quadriceps [74]. In youth female volleyball players, explosive strength also defined volleyball technique variables, specifically setting, spiking, and blocking (Can *R* = 0.64–0.80, *p* = 0.001–0.05) [71]. In a study by Valadés and colleagues [80], spike jump speed could be predicted by the player’s lower extremity power capability, determined by vertical jump height (in the presence of standing spike speed in the canonical model) at the start of the season and middle of the season (Can *R*^2^ = 0.868–0.870, *p* = 0.001). However, this finding was not present at the end of the season [80].

The association between combined body composition and anthropometric variables with technical volleyball skills in youth female ball players has been frequently investigated [69, 76–79]. A set of morphological variables, predominantly longitudinal skeleton dimensionality, as well as factors responsible for muscle to adipose tissue ratio, demonstrated significant (*β* = 0.34–0.71, *p* = 0.001–0.05) determination of block and spike jump performance; however, this finding is from a study with poor methodological quality with high ROB [69]. Similar anthropometric associations were observed by Stamm and colleagues in a female youth volleyball cohort that was reported in multiple studies [76–79]. Efficiency of offensive and defensive skills was predominantly predicted by height, weight, and indicators of muscle mass tissue in this player population [76–79].

In contrast to the associations demonstrated between body composition and volleyball skills, one study examined the association between body composition measures and soccer skills, including kicking and heading [82]. The canonical correlation between the morphological variables and the soccer-specific technical skills was not significant (Can *R*^2^ = 0.54, *p* = 0.11), indicating body composition and anthropometric attributes should not be used as predictors of soccer-specific skills in female soccer players [82].

Finally, two studies investigated the association between a variety of physical fitness attributes, including body composition, coordination, power, and speed, with handball skills in elite senior female handball players [56, 61]. One study was considered to have poor methodological quality with high ROB, which indicated female handball players with greater lower extremity explosive strength and transverse hand dimensionality achieved better results in ball manipulation, throw precision, and speed of movement with the ball (Can *R*^2^ = 0.65, *p* < 0.001) [61]. Additionally, sprinting capability was positively associated with speed of movement with the ball (Can *R*^2^ = 0.57, *p* < 0.001), and coordination/agility and upper extremity explosive strength was predictive of handball throw distance (Can *R*^2^ = 0.41, *p* < 0.001) [61]. Similarly, Čavala and colleagues [56] reported agility and explosive strength (*β* = − 0.62, *p* < 0.001), as well as greater muscle mass (*β* = 0.29, *p* < 0.001), were significant contributors to quality of handball performance. Handball performance was based on subjective assessment of both team quality and an individual player’s quality within a team [56]. Team quality was categorised into three groups: (i) elite teams of the respective age group, (ii) medium-quality teams, and (iii) low-ranking teams [56]. Individual player quality within a team was determined by the coach’s assessment of players being (i) leading team players, (ii) the remaining on-court team players and players entering the game contributing to team results, and (iii) players who rarely or never enter the game [56]. The combination of these scores resulted in a final score of 1–5 with 5 being a higher quality player (i.e. higher skilled player who is selected in higher level of competition) [56].

## Discussion

The aim of this systematic review was to determine if physical fitness attributes were associated with performance of sport-specific technical skills in female, team-based ball players. Findings revealed the physical fitness component of body composition had an association with defensive sport-specific technical skills in female, team-based ball players. Additionally, body composition was also found to have an association with throwing/shooting (using hands) sport-specific technical skills in junior participants and with movement with a ball (using hands or feet) in elite senior ball players. Finally, the physical fitness components of agility and coordination were found to be significantly associated with movement with a ball (using hands or feet) sport-specific technical skills in female, team-based ball players. The remaining physical fitness components of balance, cardiorespiratory fitness, flexibility, muscular strength, muscular endurance, power, reaction time, and speed demonstrated either no association, uncertain association, or the relationship deemed unknown with sport-specific technical skills in female, team-based ball players.

A positive relationship between body composition measures and defensive basketball technical skills in female, team-based ball players was observed in this review. In particular, successfully executing a rebound in basketball demonstrated a significant positive association with various body composition measures, including height, weight, lean muscle mass, and arm and hand span. Thus, being taller, heavier with greater muscle mass, having a larger hand span surface area, and a longer arm extension length may better enable a player to successfully reach and collect rebounds than their opposition. Similar relationships have also been demonstrated in male basketball players [95, 96]. A common strategy in basketball involves determining a player’s position based on their body size [97]. Typically, taller and heavier players are placed in a power forward or a centre position, where they can partake in gaining possession of the ball after a missed shot from their opponent or teammates (i.e. rebound) [97].

Greater height, weight, gluteal and calf girths, and lower body fat percentage were also found to have a positive relationship with better handball throwing performance, namely throwing velocity of penalty, set, and jump shot techniques, in junior players. While previous research has demonstrated an inverse relationship between body weight and motor skills in youth, these findings in reference to body weight are most likely explained by increased body fat [98, 99]. In other words, increased fat mass and obesity could lead to inefficient movement patterns, particularly when more body segments are involved [99]. Therefore, the findings in this review regarding the greater anthropometric measurements coupled with lower body fat percentage could be explained by greater lean muscle mass, particularly in the lower extremities. As the sport-specific skill of throwing or shooting a ball involves the transfer of forces via a kinetic chain, those junior ball players with increased muscle mass may be able to deliver a handball at higher velocities. However, these findings should be interpreted with caution, as the summary conclusion includes relationships from only one study [63].

Conversely to throwing, greater body composition measures, including height, weight, sum of skinfolds, arm span, and upper arm circumference, were shown to have an inverse association with the sport-specific technical skill of dribbling a soccer ball in elite senior ball players. As this technical skill requires the mastery of synchronising the movement of body segments relative to the motion of a moving ball [100], players of greater stature, body mass, and body fat percentage may demonstrate difficulty in the ability to coordinate and move their body segments in conjunction with the ball. Practically, this could result in decreased ability to maintain ball possession and advance the ball efficiently towards scoring territory during soccer games. Nevertheless, this finding should be interpreted with caution as significant relationships for elite and senior sub-analyses were drawn from only one study [89].

Conclusions from this review also revealed physical fitness attributes of agility and coordination to be significantly associated with movement with a ball (using hands or feet) sport-specific technical skill performance. In particular, change of direction speed demonstrated a relationship with dribbling a soccer ball, whereas eye-hand and whole-body coordination abilities showed relationships with running with a handball in non-match play conditions. Physical fitness qualities of agility and coordination have also been shown to have an association with dribbling a soccer ball measured outside of competitive play in male, team-based ball players [101, 102]. As such, those players who can quickly change direction in response to a stimulus and move their body segments more smoothly are able to more efficiently dribble a soccer ball in their sporting environment. This has great importance given the continual advances in the speed of play characterising successful team-based ball sports performance [103, 104], whereby fast and skilled actions (i.e. requiring high levels of agility and coordination performed at high intensity) contribute substantially to successful outcomes [101, 105]. However, it is important to note that the summary conclusion between coordination and movement with a ball (using hands or feet) was drawn from one study [55].

Systematic reviews have previously been conducted investigating the relationships between physical fitness and motor (movement) competency, or the mastery of motor skill and movement patterns that facilitates enjoyable and successful participation in physical activities, in children and adolescents [52, 98]. Strong levels of evidence support the relationships between physical fitness qualities, namely body weight, cardiorespiratory fitness, and muscular strength and endurance, with motor competency [52, 98]. Although sport participation has been demonstrated to augment motor competency [106], the majority of results from the present review indicate no association between physical fitness qualities and sport-specific technical skills in female, team-based ball players. These findings could be influenced by the fact that sport-specific technical skills are influenced by numerous constraints. In particular, sport-specific technical skills are adaptable functions of the interaction between the player, their environment, and the task [10, 107]. As such, the multifactorial nature of team-based, ball sports most likely has an influence on a player’s ability to perform a sport-specific technical skill. For example, offensive actions are typically constrained by defensive behaviours from the opponent. Given two thirds of the studies that investigated the offensive and defensive sport-specific technical skills included in this systematic review were captured during competitive play through game-related statistics, a simple physical fitness test performed in a controlled setting may be unlikely to associate with a complex sport-specific technical skill. In other words, the research design does not account for variables that support the control or emergence of an action (i.e. informational constraints [107, 108]) or representativeness of the movement performed in a particular sport context. Information variables from the task itself (e.g. rules of the sport) and the sporting environment (e.g. condition of a basketball court) interact with individual constraints (e.g. physical fitness attributes) to influence the emergence of a movement behaviour [107, 108]. Conversely, the sport-specific technical skills of throwing, kicking, and movement with a ball were primarily measured in non-match play situations. As such, many of the studies included in this review remove critical information sources that result in technical skill behaviours, thereby indicating that a holistic approach is required for understanding sport-specific technical skill performance.

The ‘no association’ findings of the present systematic review are important given that strong assumptions could be made by sporting professionals between physical fitness variables and sport-specific technical skill and its implications on talent identification, selection, and development. Therefore, it may be beneficial for sport practitioners to move away from the achievement of perfect technique by means of focusing on physical fitness, to facilitating the player’s emergence of skill by enhancing their relationship with their performance environment [107]. In other words, sport practitioners should look to provide more learning opportunities for players to explore competitive environments representative of their sport to develop emergence of functional movement patterns for technical skill performance [107]. Physical fitness characteristics are considered to be an individual constraint [107], but it is just one component and how it interacts with environmental and task constraints requires further consideration in future research, if we wish to enhance our understanding of skill adaptation. Future research designs should account for information sources used to dictate player decisions and the representativeness of movement performed in particular sport contexts, such as during competitive play.

### Strengths and Limitations

The present review incorporated an extensive search strategy and systematic screening approach [109]. This allowed the authors to identify eligible studies for inclusion in this review to address the research aim. A comprehensive critical appraisal of methodological quality of included studies with ROB assessment was performed to strengthen conclusions synthesised in the review. While a wide variety of physical fitness components and sport-specific technical skills were incorporated amongst the 41 included studies, the findings are influenced by limitations from both the literature and this review. Firstly, it is important to note that some association results between physical fitness and sport-specific technical skills measured in the 41 included studies were not reported, despite methods indicating these relationships would be investigated. These missing analyses have the potential to impact the findings from the data synthesis in the present review. Additionally, while not a direct criticism of the included studies, it was noted that some of the potential relationships were not examined (10% of total potential relationships), indicating that the data are available, however not explored, highlighting the opportunity for further investigation. Another noteworthy limitation includes nine of the summary of evidence outcomes, whether from grouped or sub-analyses, were based only on relationships from single studies. While this is not ideal, it again demonstrates the lack of research globally in female, team-based ball players. Additionally, only seven team-based ball sports were assessed within the studies eligible for inclusion in this systematic review, representing only 44% of sports included in the search strategy. Such findings further indicate a lack of research investigating the relationships between physical fitness attributes and technical skill performance in common sports played by females.

The findings synthesised in this systematic review were largely from observational studies that were cross-sectional or longitudinal in design, with only one study of experimental nature [54]. This limits the ability for conclusions to be drawn regarding causality of physical fitness attributes in relation to sport-specific technical skills. Additionally, only three studies included in this review estimated and reported statistical power, and another 22% of studies were classified as having poor methodological quality or high ROB, highlighting the lack of high-quality evidence in female, team-based ball sport research.

An additional limitation was the authors’ decision to only include articles published in the English language which meant that some relevant empirical literature may have been missed. Lastly, only physical fitness attributes were included in the search strategy in this review, thereby examining just one of the many constraints that can influence performance of a sport-specific technical skill. Future reviews could consider multiple variables that may potentially influence the performance of such skills, for instance, decision-making abilities, dynamics of the competitive environment, and psychological factors, such as emotions and confidence.

## Conclusion

The present systematic review found evidence to show that in female, team-based ball players, a relationship exists between (i) defensive sport-specific technical skills and body composition, (ii) movement with a ball and agility, and (iii) movement with a ball and coordination. Additionally, body composition was found to be associated with movement with a ball in elite senior ball players and with throwing/shooting (using hands) in junior ball players. These findings may assist team sport practitioners with insight into continued areas for development to improve technical skill capacity. Specifically, practitioners could develop body composition, agility, and coordination fitness to further develop ball skills during represented training tasks. Most physical fitness measures were not associated with sport-specific technical skills in female, team-based ball players. Findings indicate that there is also limited, high-quality evidence available to demonstrate relationships between physical fitness qualities and sport-specific technical skills in female, team-based ball players. The lack of associations is possibly due to the reductionist methods and reporting in the available empirical literature and limited research examining a holistic approach of sport-specific technical skills. These findings may provide insight for team sport practitioners partaking in talent identification and development programs to consider the collective interaction of influencing factors on sport-specific technical skill performance, rather than solely physical fitness performance results. Additionally, a lack of research exists investigating the relationships between physical fitness attributes and sport-specific technical skill performance in female players globally. High-quality, holistic evidence, including a wider range of team-based ball sports, is needed to better understand the relationship and the role that physical fitness plays in the multifactorial nature of sport-specific technical skills performance in female ball players.

## Supplementary information

**Additional file 1:.** Online Resource 1: The finalised MEDLINE search strategy.

**Additional file 2:.** Online Resource 2: Key data extracted for this review.

## Data Availability

Not applicable.

## Abbreviations

MeSH: Medical subject headings
PRISMA: Preferred reporting items for systematic reviews and meta-analyses
ROB: Risk of bias
