# Supplementary material for: The Relationship Between Physical Fitness Qualities and Sport-Specific Technical Skills in Female, Team-Based Ball Players: A Systematic Review
Source: Sports Med Open. 2020 Apr 15;6:18. doi: 10.1186/s40798-020-00245-y (PMC7158966; doi:10.1186/s40798-020-00245-y)
Supplement: Supplementary file 1 — Additional file 1:. Online Resource 1: The finalised MEDLINE search strategy. [file 40798_2020_245_MOESM1_ESM.docx]

**Online Resource 1**

MEDLINE search strategy used for the systematic review titled, “The relationship between physical fitness qualities and sport-specific technical skills in female, team-based ball players: A systematic review” submitted to Sports Medicine - Open.

Title: The relationship between physical fitness qualities and sport-specific technical skills in female, team-based ball players: A systematic review

Journal: Sports Medicine - Open

Authors: Jessica B. Farley^1^, Joshua Stein^1^, Justin W. L. Keogh^1,2,3,4^, Carl T. Woods^5^, Nikki Milne^1^

^1^ Faculty of Health Sciences and Medicine, Bond Institute of Health and Sport, Bond University, Australia

^2^ Sports Performance Research Centre New Zealand, AUT University, Auckland, New Zealand

^3^ Cluster for Health Improvement, Faculty of Science, Health, Education and Engineering, University of the Sunshine Coast, Australia

^4^ Kasturba Medical College, Mangalore, Manipal Academy of Higher Education, Manipal, Karnataka, India

^5^ Institute for Health and Sport, Victoria University, Melbourne, Australia

Corresponding author: Jessica B. Farley, Email: jfarley@bond.edu.au

1. female/
2. female*.af
3. women/
4. wom#n.af
5. girl*.af
6. 1 OR 2 OR 3 OR 4 OR 5
7. athletes/
8. athlet*.af
9. player*.af
10. 7 OR 8 OR 9
11. 6 AND 10
12. ball sport*.af
13. ball game*.af
14. ballgame*.af
15. basketball/
16. basketball.af
17. football/
18. football.af
19. soccer/
20. soccer.af
21. rugby.af
22. volleyball/
23. volleyball.af
24. cricket.af
25. baseball/
26. baseball.af
27. softball.af
28. handball.af
29. netball.af
30. oztag.af
31. futsal.af
32. 12 OR 13 OR 14 OR 15 OR 16 OR 17 OR 18 OR 19 OR 20 OR 21 OR 22 OR 23 OR 24 OR 25 OR 26 OR 27 OR 28 OR 29 OR 30 OR 31
33. 11 AND 32
34. physical fitness/
35. fitness.af
36. physical endurance/
37. endurance.af
38. cardiovascular.af
39. cardiorespiratory.af
40. aerobic.af
41. anaerobic threshold/
42. anaerobic.af
43. ventilatory threshold.af
44. ventilator thresholds.af
45. oxygen consumption/
46. oxygen consumption.af
47. muscle strength/
48. strength.af
49. force.af
50. power.af
51. anthropometry/
52. anthropometr*.af
53. body height/
54. height.af
55. body weight/
56. weight.af
57. body mass index/
58. mass.af
59. body weights measures/
60. Body Composition/
61. composition.af
62. body size.af
63. body fat.af
64. muscle.af
65. bone density/
66. bone.af
67. musculoskeletal development/
68. development.af
69. growth.af
70. flexibility.af
71. extensibility.af
72. muscle length.af
73. range of motion articular/
74. range of motion.af
75. joint range.af
76. joint mobility.af
77. joint hypomobility.af
78. joint stiffness.af
79. joint hypermobility.af
80. joint instability/
81. laxity.af
82. psychomotor performance/
83. psychomotor performance.af
84. motor performance.af
85. motor ability.af
86. motor skills/
87. motor skill*.af
88. coordination.af
89. motor competenc*.af
90. motor proficienc*.af
91. motor development.af
92. motor activity/
93. motor activity.af
94. fine motor.af
95. gross motor.af
96. fundamental ADJ3 movement.af
97. fundamental ADJ3 skill.af
98. movement/
99. movement.af
100. muscular sense.af
101. proprioception/
102. proprioception.af
103. kinesthesis/
104. kinesthesia.af
105. dexterity.af
106. precision.af
107. postural balance/
108. balance.af
109. control.af
110. body equilibrium.af
111. speed.af
112. agility.af
113. reaction time.af
114. change of direction.af
115. run*.af
116. sprint*.af
117. jump*.af
118. throw*.af
119. catch*.af
120. strik*.af
121. kick*.af
122. profil*.af
123. characteristic*.af
124. physical.af
125. physiological.af
126. exercise test/
127. test*.af
128. measur*.af
129. evaluation.af
130. 34 OR 35 OR 36 OR 37 OR 38 OR 39 OR 40 OR 41 OR 42 OR 43 OR 44 OR 45 OR 46 OR 47 OR 48 OR 49 OR 50 OR 51 OR 52 OR 53 OR 54 OR 55 OR 56 OR 57 OR 58 OR 59 OR 60 OR 61 OR 62 OR 63 OR 64 OR 65 OR 66 OR 67 OR 68 OR 69 OR 70 OR 71 OR 72 OR 73 OR 74 OR 75 OR 76 OR 77 OR 78 OR 79 OR 80 OR 81 OR 82 OR 83 OR 84 OR 85 OR 86 OR 87 OR 88 OR 89 OR 90 OR 91 OR 92 OR 93 OR 94 OR 95 OR 96 OR 97 OR 98 OR 99 OR 100 OR 101 OR 102 OR 103 OR 104 OR 105 OR 106 OR 107 OR 108 OR 109 OR 110 OR 111 OR 112 OR 113 OR 114 OR 115 OR 116 OR 117 OR 118 OR 119 OR 120 OR 121 OR 122 OR 123 OR 124 OR 125 OR 126 OR 127 OR 128 OR 129
131. 33 AND 130
132. Task Performance Analysis/
133. psychomotor performance/
134. motor skills/
135. skill*.af
136. athletic performance/
137. performance.af
138. success.af
139. qualit*.af
140. abilit*.af
141. 132 OR 133 OR134 OR 135 OR 136 OR 137 OR 138 OR 139 OR 140
142. kick*.af
143. pass*.af
144. dribbl*.af
145. shoot*.af
146. spik*.af
147. tackl*.af
148. turn*.af
149. handball*.af
150. catch*.af
151. receiv*.af
152. trap*.af
153. field*.af
154. serv*.af
155. hit*.af
156. head*.af
157. block*.af
158. defens*.af
159. defend*.af
160. offens*.af
161. offend*.af
162. attack*.af
163. lift*.af
164. jump*.af
165. run*.af
166. throw*.af
167. pitch*.af
168. bowl*.af
169. strik*.af
170. bunt*.af
171. sprint*.af
172. ball velocity.af
173. ball speed.af
174. accuracy.af
175. sport specific.af
176. acceleration/
177. acceleration.af
178. sport specific.af
179. 142 OR 143 OR 144 OR 145 OR 146 OR 147 OR 148 OR 149 OR 150 OR 151 OR 152 OR 153 OR 154 OR 155 OR 156 OR 157 OR 158 OR 159 OR 160 OR 161 OR 162 OR 163 OR 164 OR 165 OR 166 OR 167 OR 168 OR 169 OR 170 OR 171 OR 172 OR 173 OR 174 OR 175 OR 176 OR 177 OR 178
180. 141 AND 179
181. 131 AND 180
182. *Limit to English language*
