# Supplementary material for: The Relationship Between Physical Fitness Qualities and Sport-Specific Technical Skills in Female, Team-Based Ball Players: A Systematic Review
Source: Sports Med Open. 2020 Apr 15;6:18. doi: 10.1186/s40798-020-00245-y (PMC7158966; doi:10.1186/s40798-020-00245-y)
Supplement: Supplementary file 2 — Additional file 2:. Online Resource 2: Key data extracted for this review. [file 40798_2020_245_MOESM2_ESM.docx]

**Online Resource 2**

Characteristics of studies included in the systematic review titled, “The relationship between physical fitness qualities and sport-specific technical skills in female, team-based ball players: A systematic review” submitted to Sports Medicine – Open.

Title: The relationship between physical fitness qualities and sport-specific technical skills in female, team-based ball players: A systematic review

Journal: Sports Medicine - Open

Authors: Jessica B. Farley^1^, Joshua Stein^1^, Justin W. L. Keogh^1,2,3,4^, Carl T. Woods^5^, Nikki Milne^1^

^1^ Faculty of Health Sciences and Medicine, Bond Institute of Health and Sport, Bond University, Australia

^2^ Sports Performance Research Centre New Zealand, AUT University, Auckland, New Zealand

^3^ Cluster for Health Improvement, Faculty of Science, Health, Education and Engineering, University of the Sunshine Coast, Australia

^4^ Kasturba Medical College, Mangalore, Manipal Academy of Higher Education, Manipal, Karnataka, India

^5^ Institute for Health and Sport, Victoria University, Melbourne, Australia

Corresponding author: Jessica B. Farley, Email: jfarley@bond.edu.au

Online Resource 2: Characteristics of included studies

| Study Author (Year) | Female Athlete Participant Characteristics | Physical Fitness Measure(s) Investigated | | Sport-Specific Technical Skill Outcome(s) | Main Findings |
| --- | --- | --- | --- | --- | --- |
| HANDBALL | | | | | |
| Bojić & Pavlović (2015) [55] | School youth  (n = 60) | Eight with bending, sideways steps, dribbling with one hand, bat coordination, receiving and throwing the ball from the sitting position, sideway vaults, jumping over a horizontal rope | | Handball shooting precision, speed of movement with handball, speed of movement with and without the ball with shooting on goal | Canonical correlation analysis revealed statistically significant relationships (-0.33 to 0.25, p = 0.05-0.00) between some of the agility and coordination abilities and the handball technical skills. |
| Čavala et al. (2008) [56] | Elite senior  (n = 53) | Height, weight, anthropometric measurements, skinfolds, side-steps, eight with bending, shuttle-run test, hand/foot tapping, standing long/high/triple jumps, 2-kg medicine ball throws (3 positions), 20/30/40-m sprints | | Handball performance | Regression analysis indicated the handball performance to be predominantly determined by agility and explosive strength (ß = -0.62, p < 0.001), as well as muscle mass (ß = 0.29, p < 0.001). |
| Eriksrud et al. (2019) [57] | Elite senior  (n = 13) | HSEBT | | Handball throwing velocity and accuracy | Pearson correlation analysis concluded no significant correlations were found between HSEBT reaches and handball throwing velocity. Athletes with greater HSEBT reaches correlated with lower throwing accuracy (r = 0.622-0.839, p = 0.001-0.041). |
| Granados et al. (2008) [58] | Elite senior  (n = 16) | Height, body mass, % body fat, BMI, fat-free mass, submaximal intermittent running test, 1-RM bench press, vertical jump, power-load relationships bench press and squat, 15-m sprint | | Handball throwing velocity | Pearson correlation analysis revealed the individual standing throw velocity values correlated (p < 0.05) with the individual values of concentric power production at the load of 45% 1RM (bench press) (r = 0.61) and with the individual values of concentric power production at the load of 80% BM (r = 0.65) during parallel squat action (at time point 3). Statistically significant correlations were also observed from T2 to T3 between standing throwing velocity changes and relative changes in velocity at 100% BMsquat (r = 0.66, P< 0.05) and between changes in three-step throwing velocity and 70% 1RM bench press (r = 0.64, P < 0.05). |
| Granados et al. (2013) [59] | Elite senior national  (n = 16)  Elite senior international  (n = 14) | 1-RM bench press, power-load relationships bench press and squat | | Handball throwing velocity | Throwing velocity in international-level female handball players depends more on the capabilities of the upper (r = 0.72, p < 0.05) and lower extremity (r = 0.59-0.71, p < 0.05) to produce maximal power with submaximal loads than in lower-level female handball players determined by Pearson correlation analysis. |
| Hoff & Almåsbakk (1995) [54] | Elite senior  (n = 16) | 1-RM bench press | | Handball throwing velocity | A significant correlation (Spearman rank) was found between handball throwing velocity and 1-RM bench press (r = 0.883, p<0.05) after a training period. |
| Karadenizli (2016) [60] | Elite senior  (n = 54) | Height, Weight, Anthropometric measurements, BMI, CMJ | | Handball throwing velocity | Weak-to moderate significant associations were shown by Spearman correlations between handball throwing velocity and body height (r = 0.393; p = 0.003), weight (r = 0.397; p = 0.003), hand length (r = 0.391; p = 0.003), and arm span (r = 0.462; p < 0.001). |
| Katić et al. (2007) [61] | Elite senior  (n = 53) | Height, weight, anthropometric measurements, skinfolds, Side-steps, eight with bending, shuttle-run test, hand/foot tapping, standing long/high/triple jumps, 2-kg medicine ball throws, 20/30/40-m sprints | | Handball throwing precision and distance, speed of movement with/without ball, ball manipulation | Canonical correlation analysis revealed players with above-average explosive strength and above-average transverse hand dimensionality achieved above-average results in specific ability of ball manipulation, throw precision, and speed of movement with ball (Can R^2^ = 0.65, p < 0.001). |
| McGhie et al. (2018) [62] | Elite senior  (n = 20) | 1-RM seated leg press, CMJ (unilateral and bilateral; no arm swing) | | Handball jump throw height | Impulse (35% 1-RM) (r = 0.52, p = 0.020) and relative impulse at 200-ms (35% 1-RM) (r = 0.60, p = 0.005) showed a significant Pearson’s correlation with jump throw jump height. |
| Moss et al. (2015) [63] | Elite youth  (n = 73)  Non-elite youth  (n = 47) | Height, body mass, skinfolds,  anthropometric measurements, waist-to-hip ratio, % body fat | | Handball throwing velocity | Pearson correlation analysis revealed body composition attributes to have significantly positive (r = 0.23-0.65, p < 0.001-0.02) and negative (r = -0.34 to -0.29, p < 0.001-0.003) relationships with handball throwing velocity of various throw types. |
| Piscitelli et al. (2016) [64] | Elite senior  (n = 24) | Height, body mass, BMI,  anthropometric measurements, whole-body and regional body composition | | Handball throwing velocity | Trunk BMC and right leg BMC consistently correlated with ball-throwing velocity (r = 0.45-0.56 and 0.46-0.52, respectively) revealed by Pearson correlation analysis. |
| Saavedra et al. (2018) [65] | Elite senior and junior  (n = 80) | Height, mass, BMI, Yo-Yo IR2, hand dynamometry  CMJ, medicine ball throw, 10- and 30-m sprints | | Handball throwing velocity | Handball throwing velocity was correlated (Pearson correlation analysis) with stature, mass, power, and speed (r = 0.367-0.533, p = 0.001-0.05). |
| Schwesig et al. (2016) [66] | Elite senior  (n = 20) | Height, body mass, BMI, arm strength, arm flexibility | | Handball throwing velocity | Pearson correlation analysis revealed moderate correlations between handball throwing velocity and elbow flexion ROM (r = -0.540, p < 0.05), GIRD (r = 0.448, p < 0.05), and shoulder retroversion strength (r = 0.431-0.521, p < 0.05). |
| van den Tillaar & Ettema (2004) [67] | Elite senior  (n = 20) | Height, body mass, skinfolds, anthropometric measurements, fat-free mass, isometric arm strength | | Handball throwing velocity | Pearson correlations were found between maximal isometric arm strength and throwing velocity (r = 0.49, p = 0.027). |
| Wagner et al. (2019) [68] | Elite senior  (n = 10) | Incremental treadmill-running test, isokinetic trunk rotation and shoulder IR strength, isometric leg extension strength, CMJ (with arm swing), 15- and 30-m sprints | | Handball throwing velocity, handball throwing jump height, game-based performance test (offense and defence time) | Relationship results via Pearson correlation analysis include: defence time and 30-m sprint time (r = 0.55, p = 0.008); jump height in the jump shot and CMJ (r = 0.71, p < 0.001); and ball velocity in the jump shot and shoulder IR torque (r = 0.84, p < 0.001). |
| VOLLEYBALL | | | | | |
| Grgantov et al. (2007) [69] | Non-elite youth  (n = 246) | Standing reach, height, weight, anthropometric measurements, skinfolds | | Volleyball game-related performance measures | Regression correlation analysis demonstrated performance in spike and block technique variables are predominantly determined by longitudinal skeleton dimensionality (ß = 0.31-0.65, p < 0.001-0.05) (particularly in 14-17 years age groups) and quality of muscle tissue (ß = -0.35 to -0.24, p < 0.05) (16-17-year-old athletes). |
| Ikeda et al. (2018) [70] | Collegiate  (n = 17) | Standing long jump, vertical jump | | Volleyball spike jump height | Positive correlations (Pearson) were found between spike jump height and vertical jump height (r = 0.495, p < 0.05) and spike jump height and standing long jump distance (r = 0.708, p < 0.01). |
| Katić et al. (2006) [71] | Non-elite youth  (n = 197) | 6x6 run, 9-3-6-3-9-m run, T-test, hexagon test, hand/foot tapping, standing long jump, standing and running vertical jump, 1-kg medicine ball throw | | Volleyball game-related performance measures | The canonical factor isolated from the set of motor variables was predominantly defined by force regulation to determine volleyball technical efficiency (specifically, setting, spike, and block) (Can R = 0.64-0.80, p = <0.001-0.05). |
| Melrose et al. (2007) [72] | Non-elite youth  (n = 29) | Height, weight, BMI, skinfolds, % body fat, anthropometric measurements, grip strength, isometric maximal leg strength, sit-up test, sit-and-reach, shoulder rotation test, T-test, 9.14-m shuttle test, stork stand balance, vertical jump, standing broad jump | | Volleyball spike and serve velocity | A strong correlation was found between serving velocity and isometric hand grip strength (r = 0.60, p < 0.05). A moderate correlation was found with serving velocity and weight (r = 0.48, p < 0.05) and lean body mass (r = 0.58, p < 0.05). Spiking velocity had a strong correlation with the stork stand (r = 0.60, p < 0.05). |
| Mielgo-Ayuso et al. (2015) [73] | Elite senior  (n = 42) | Height, body mass, BMI, skinfolds, anthropometric measurements, | | Volleyball spike jump | Pearson correlation analysis demonstrated significant weak-to-very strong correlations between spike jump measures and body composition physical fitness attributes (r = 0.339-0.879, p < 0.001-0.05). |
| Sattler et al. (2015) [74] | Elite senior  (n = 82) | Knee extensors and flexors strength | | Volleyball block-jump and spike-jump height | Discriminant canonical analysis demonstrated relative isokinetic strength variables are more strongly related to block-jump performance than to spike-jump performance (Can R = 0.59 and 0.46, respectively, p < 0.001). |
| Sattler et al. (2016) [75] | Elite senior  (n = 67) | Height, body mass, knee extensors and flexors strength | | Volleyball block-jump and spike-jump height | Multiple regression revealed isokinetic variables were found to be more valid predictor of block jump (R^2^ = 0.42, p = 0.01) than spike jump (R^2^ = 0.39, p = 0.01). |
| Stamm (2004) [76] | Non-elite youth  (n = 46) | Height, weight, anthropometric measurements, skinfolds | | Volleyball game-related performance measures | Linear prediction models composed of 14 body measurements significantly correlated with game performance (R^2^ = 0.32-0.83, p < 0.05). |
| Stamm et al. (2005) [77] | Elite youth  (n = 32) | Height, weight, anthropometric measurements, skinfolds, auditory reaction, visual reaction | | Volleyball game-related performance measures | Linear regression analysis revealed 14 of the 49 anthropometric variables and seven reaction time tests correlated with game proficiency (R^2^ = 0.32-0.98, p < 0.05). |
| Stamm et al. (2001) [78] | Non-elite youth  (n = 33) | Height, weight, anthropometric measurements, skinfolds | | Volleyball game-related performance measures | The preconditions for volleyball proficiency (R^2^ = 0.32-0.83, p < 0.05) are a certain size in weight and height, trunk (chest, waist, and hip) circumferences, extremity circumferences, and wrist breadth according to linear regression analysis. |
| Stamm et al. (2003) [79] | Non-elite youth  (n = 32) | Height, weight, anthropometric measurements, skinfolds, 20-m shuttle run, sit-up test, sit-and-reach, zigzag run test, standing and running vertical jumps, medicine ball throw test, auditory reaction, visual reaction | | Volleyball game-related performance measures | Body characteristics are important for all the main elements of the game, determining performance efficiency within 32-83%. The 20-m shuttle run, sit-and-reach, zigzag run test, medicine ball throw, and reaction time tests showed significant correlation with game proficiency (R^2^ = 0.18-0.44, p < 0.05) deemed by linear regression analysis. |
| Valadés et al. (2016) [80] | Elite senior  (n = 11) | Reach height, height, body mass, skinfolds, BMI, 1-RM bench press and pullover, running vertical jump, medicine ball throws | | Volleyball standing and jump spike speed | Multiple regression demonstrated vertical jump height to be included in the prediction model for jump spike speed for two out of three time points during the season (ß = 0.43-0.89, p < 0.001-0.01), in conjunction with standing spike speed (ß = 0.65-0.73, p < 0.001) and body height (ß = -0.40, p = 0.02) (one time period only). |
| SOCCER |  |  |  |  |  |
| Brooks et al. (2013) [81] | Collegiate  (n = 22) | Skinfolds, VO_2_max, 1-RM squat parallel test, knee and hip torque, Illinois agility test, vertical jump, 40-yard dash, 100-m sprint | | Soccer kicking velocity | Ball velocity demonstrated very strong relationships with vertical jump (r = 0.91, p < 0.05) and knee torque (r = 0.93, p < 0.05) via Pearson correlation analysis. |
| Jelaska et al. (2015) [82] | Elite senior  (n = 70) | Height, weight, anthropometric measurements, skinfolds | | Soccer kicking precision and distance, soccer heading precision and distance, ball handling speed, soccer dribbling speed | Results of the canonical correlation analysis indicated that the conditionality of specific motor variables based on the dimensions of morphological status is weak and not significant (Can R^2^ = 0.54, p = 0.11). |
| Kaminski et al. (2007) [83] | Collegiate  (n = 21)  School youth  (n = 26) | Modified Romberg test | | Soccer headers | Pearson correlation results indicated that there were no significant correlations between the total number of game headers and the change in balance scores from preseason to postseason in both female soccer player groups (r = -0.363 to 0.268, p = 0.106-0.943). |
| Kutlu et al. (2017) [84] | Collegiate  (n = 34) | T-drill, Illinois agility run, change of direction and acceleration test, 20-m sprint | | Agility and Skill test (soccer ball skills and goal success) | The Agility and Skill test demonstrated significant moderate relationships with the 20-m sprint (r = 0.49-0.52, p < 0.001) and weak-to-moderate associations with all agility tests (r = 0.37-0.56, p < 0.001-0.03) deemed by Pearson correlation analysis. |
| Mujika et al. (2009) [85] | Elite senior  (n = 17)  Elite youth  (n = 17) | Height, body mass, skinfolds, Yo-Yo IR1, 15-m agility run, CMJ (with and without arm swing), 15-m sprint | | 15-m soccer ball dribbling slalom course | Pearson correlation analysis revealed a significant relationship between agility and soccer ball dribbling (r = 0.61, p < 0.001). |
| Perroni et al. (2018) [86] | Non-elite youth  (n = 16) | Height, weight, BMI | | Soccer ball juggling, dribbling, passing, shooting, and heading | Large significant correlations (p<0.05) were found by Spearman analysis between anthropometric attributes and juggling (r = -0.66 to 0.55) and headers (r = 0.54-0.58). |
| BASKETBALL | | | | | |
| Dyer et al. (2018) [87] | Elite senior  (n = 64) | Second to fourth digit ratio | | Basketball game-related performance measures | Players with lower 2D:4D ratios tended to perform better statistically in basketball games, especially defensively in terms of accumulating more blocks and rebounds, and being more efficient scorers, irrespective of their age and body size (approximate r = -0.20 to -0.40; data reported in Figure 1 in Dyer et al. (2018); raw data not published). |
| Fort-Vanmeerhaeghe et al. (2016) [88] | Elite youth  (n = 23) | Yo-Yo IR1, "Suicide" run test, bench press and leg press 1-RM, T-test, squat jump, CMJ, Abalakov jump, overhead medicine ball throw, 3/4 basketball court sprint test, repeated sprint ability test | | Basketball game-related performance measures | The most significant relationships were noted  between assists and steals and aerobic and  anaerobic power, as well as speed and agility  performance measures (r = -0.701 to 0.661, p < 0.01-0.05) by Pearson correlation analysis. |
| Garcia-Gil et al. (2018) [89] | Elite senior  (n = 41) | Body mass, body height, BMI, skinfolds, arm/ thigh/lower leg circumferences, arm span, T-Drill test, CMJ (with arm swing), 20-m sprint | | Basketball dribbling test, basketball game-related performance measures | Pearson or Spearman correlation analysis revealed various body composition variables were associated with basketball dribbling and player performance (r = 0.320-0.742, p < 0.05-0.005). |
| Ramos et al. (2019) [90] | Elite youth  (n = 192) | Body mass, stature, sitting height, skinfolds, arm span, hand span, relative fat mass %, absolute fat-free mass, BMI, handgrip strength, sit ups, sit and reach,  T-test, CMJ (with and without arm swing), 2-kg medicine ball throw, 20-m sprint | | Basketball game-related performance measures | Pearson or Spearman correlation analyses demonstrated significant (p < 0.01-0.05) relationships between health- (anthropometric, strength) (r = 0.148-0.367) and performance-related (agility, power, speed) (r = -0.214 to 0.255) physical fitness attributes with basketball game-related performance measures. |
| NETBALL |  |  |  |  |  |
| Elliot et al. (1983) [91] | Elite senior  (n = 12) | Standing and sitting height, weight, anthropometric measurements, skinfolds (% body fat), knee/ elbow/wrist/grip strength, shoulder and wrist flexibility | | Netball shooting accuracy | Wrist flexibility, relative sitting height, and upper extremity length provide the best prediction of netball shooting accuracy rating (multiple correlation = 0.93). Wrist flexibility demonstrated the only significant strong correlation (r = 0.686, p < 0.05) with netball shooting accuracy. |
| Tissera et al. (2019) [92] | School youth  (n = 45) | Standing stature, sitting stature, body mass, Yo-Yo IR1, 505 agility test, CMJ, seated medicine ball throw, 20-m sprint | | Netball game related performance measures | Skill-based success in catching and passing events consistently demonstrated moderate correlations (Pearson) with physical fitness (r = 0.460-0.541, p = 0.001-0.005). |
| LACROSSE |  |  |  |  |  |
| Marsh et al. (2010) [93] | Collegiate  (n = 15) | Grip strength, Biodex Stability System, | | Lacrosse shot accuracy and velocity | A strong correlation (Pearson) was significant between lacrosse-shot error and one difficulty level on the Biodex Stability System [BSS L8 eyes open (r = 0.760, p = 0.011)]. |
| SOFTBALL |  |  |  |  |  |
| Pugh et al. (2001) [94] | Experienced pitchers  (n = 16) | Leg, arm, and grip strength | | One-step windmill underhand throw speed | Multiple regression revealed that useful predictors of ball speed include arm and grip strength in experienced underhand pitchers (r_p_ = 0.60-0.65, p< 0.05). |

Abbreviations: 1-RM = one-repetition maximum; 2D:4D = digit ratio between second and fourth digits; BMI = body mass index; BMC = bone mineral content; BMD = bone mineral density; BSS = Biodex Stability System; CMJ = countermovement jump; GIRD = glenohumeral internal rotation deficit; HSEBT = hand reach star excursion balance test; r = correlation; r_p_ = partial correlation_;_ ROM = range of motion; Yo-Yo IR1 = Yo-Yo Intermittent Recovery Level 1 test; Yo-Yo IR2 = Yo-Yo Intermittent Recovery Level 2 test.
